# Early Aortic Valve Replacement vs. Conservative Management in Asymptomatic Severe Aortic Stenosis Patients With Preserved Ejection Fraction: A Meta-Analysis

**DOI:** 10.3389/fcvm.2020.621149

**Published:** 2021-02-03

**Authors:** Tan Yuan, Yi Lu, Chang Bian, Zhejun Cai

**Affiliations:** ^1^Department of Cardiology, The Second Affiliated Hospital, Zhejiang University School of Medicine, Hangzhou, China; ^2^Jiaxing Key Laboratory of Cardiac Rehabilitation, Jiaxing, China

**Keywords:** asymptomatic, aortic stenosis, aortic valve replacement, conservative treatment, preserved ejection fraction

## Abstract

**Background:** Aortic stenosis (AS) is the most common valvular disease in developed countries. Until now, the specific timing of intervention for asymptomatic patients with severe aortic stenosis and preserved ejection fraction remains controversial.

**Methods:** A systematic search of four databases (Pubmed, Web of science, Cochrane library, Embase) was conducted. Studies of asymptomatic patients with severe AS or very severe AS and preserved left ventricular ejection fraction underwent early aortic valve replacement (AVR) or conservative care were included. The end points included all-cause mortality, cardiac mortality, and non-cardiac mortality.

**Results:** Four eligible studies were identified with a total of 1,249 participants. Compared to conservative management, patients who underwent early AVR were associated with lower all-cause mortality, cardiac mortality, and non-cardiac mortality rate (OR 0.16, 95% CI 0.09–0.31, *P* < 0.00001; OR 0.12, 95% CI 0.02–0.62, *P* = 0.01; OR 0.36, 95% CI 0.21–0.63, *P* = 0.0003, respectively).

**Conclusions:** Early AVR is preferable for asymptomatic severe AS patients with preserved ejection fraction.

## Introduction

Aortic stenosis (AS) is the most common valvular disease in developed countries, which affects 5% of population >65 years and 3% of population over 75 (1–3). Degenerative process is the major etiology of AS, and ultimately leads to the valve remodeling and systemic blood flow restriction (4). The onset of symptoms (angina, dyspnea on exertion, and syncope) heralds a poor prognosis (5). For symptomatic AS patients, the annual mortality is close to 25% and the average survival time is only 2–3 years (6). Therefore, aortic valve replacement (AVR), either surgical or interventional, is strongly recommended, which is the current only feasible treatment for symptomatic AS (7, 8).

However, ~50% of patients with severe AS are asymptomatic at the time of diagnosis (3, 9). The mortality in asymptomatic patients without surgery ranges widely across the studies and the cumulative 5-year all-cause death incidences is up to 62% (10–13). However, the potential benefit of AVR for asymptomatic patients with severe AS may not outweigh the operative complications (14). It had been reported that operative mortality of isolated AVR for AS was 1–3% in patients <70 years old and 3–8% in senior patients (14). According to the current guidelines, AVR is recommended for asymptomatic AS patients with reduced left ventricular ejection fraction (LVEF) (7).

On the other hand, treatment for asymptomatic severe AS with preserved LVEF is still debatable. The recent guidelines suggest a “watching waiting” strategy for the remaining majority of asymptomatic patients (7). This recommendation was based on non-randomized studies (12, 15) with low evidence levels (16). Several recent studies reported that early AVR for severe asymptomatic AS was associated lower mortality and hospitalization for heart failure at 5-year of follow-up with improved long-term outcomes (13). To facilitate clinical decision-making, we conducted this meta-analysis to compare the outcomes of early AVR and conservative strategy in asymptomatic AS patients with preserved LVEF.

## Patients and Methods

The review was reported according to the Preferred Reporting Items for Systematic Reviews and Meta-Analyses statement standards.

### Searching Strategy

We searched four databases: Pubmed, Web of Science, Embase, and Cochrane library. The last search was performed on July 29, 2020. We used search terms as follows: “asymptomatic” and “aortic stenosis”. There were no language and year of publication constraints in literature search. The detailed searching strategy is provided in the online Supplementary File.

### Eligibility Criteria and Study Selection

We included both randomized controlled trials (RCTs) and non-randomized studies if they met the following criteria: (1) patients with severe AS or very severe AS [severe AS was defined as an aortic valve area ≤1 cm^2^ or peak aortic velocity ≥4 m/s or mean transaortic pressure gradient ≥40 mmHg; very severe AS was defined as an aortic-valve area of ≤0.75 cm^2^ with either an aortic jet velocity of ≥4.5 m/s or a mean transaortic gradient of ≥50 mmHg]; (2) asymptomatic (without symptoms like exertional angina pectoris, syncope, exertional dyspnea, etc.); (3) LVEF ≥50% or preserved LVEF (LVEF was assessed by transthoracic echocardiography); and (4) included the outcomes of patients underwent early AVR procedure and patients received conservative care (early AVR procedure was defined as intervention before the onset of symptoms or declined LVEF). We excluded abstracts, reviews, case reports, meeting abstracts, and editorial material. Eligibility screening was conducted with a two-step strategy (title/abstract screening and full-text screening). Two independent reviewers screened all potentially eligible studies.

### Data Extraction and Risk of Bias in Included Studies

Two independent authors extracted the relevant data. The extracted data included: (1) time, region and design; (2) study period; (3) follow-up period; (4) number of patients; (5) mean age and gender ratio of participants; and (6) LVEF, aortic valve area.

**Figure 1:**
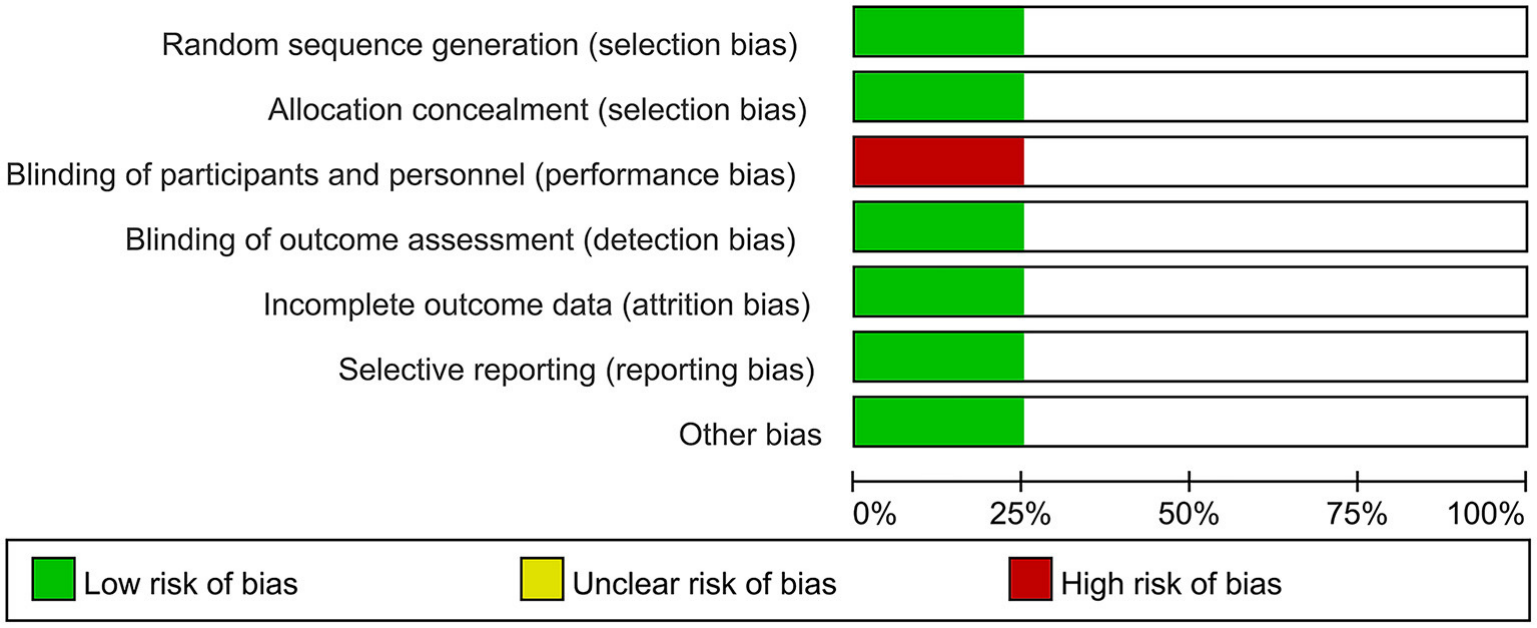
Bias assessment for included RCT trials.

**Figure 2:**
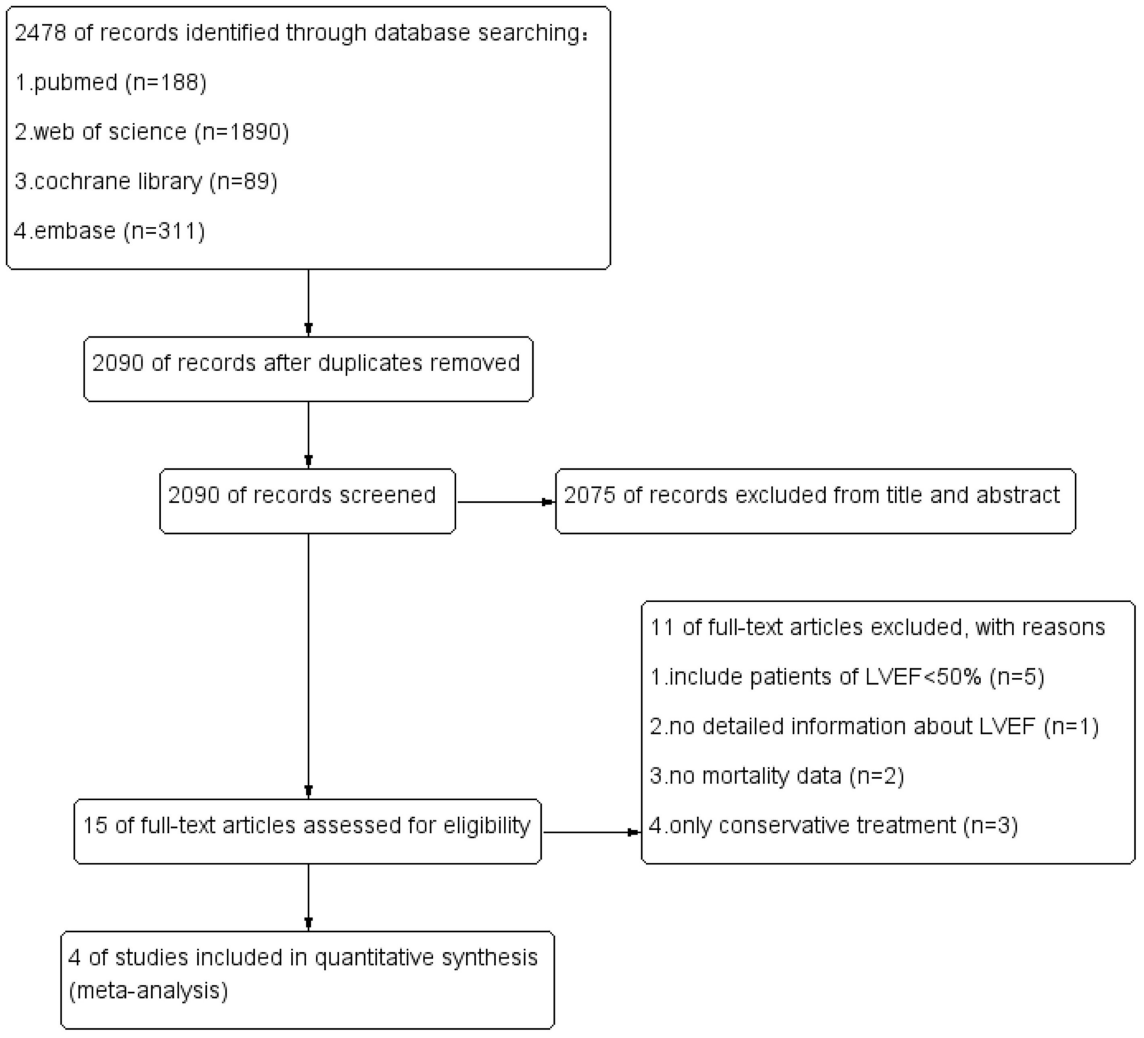
Study selection process.

Two independent reviewers assessed the risk of bias among included RCT trials by Cochrane Risk of Bias tool. The risk of bias in included non-RCT studies was assessed by the Newcastle Ottawa scale. No disagreements arose in the quality assessment. Quality assessment of all included studies indicated low risk of bias. The overall risk of bias was graded as low risk. The results are enclosed as Table 1, Figure 1. Publication bias was assessed using funnel plots (Figure 2), which showed no evidence of publication bias.

**Table 1:** Newcastle-Ottawa scale.

**Study**	**Selection**	**Comparability**	**Outcomes**	**Quality score**
Kang	⋆⋆⋆⋆	⋆⋆	⋆⋆	8
Kim	⋆⋆⋆⋆	⋆⋆	⋆⋆	8
Bohbot	⋆⋆⋆⋆	⋆⋆	⋆⋆	8

### Data Items

We sought data according to the following PICOS: P (Population), patients with asymptomatic severe asymptomatic AS (LVEF ≥50%); I (Intervention), early AVR; C (Comparison), conservative strategy; O (Outcome), all-cause mortality, cardiac mortality, and non-cardiac mortality; and S (Study type), RCT, and observational studies.

### End Points

The primary end points were all-cause mortality and cardiac mortality. Secondary end point was non-cardiac mortality. This meta-analysis compared the prognosis of asymptomatic AS patients with preserved EF underwent early AVR procedure with those who received conservative care strategy.

### Statistical Analysis

We used odd ratios (ORs), hazard ratio (HR), and 95% confidence intervals (95% CIs) to serve as primary index statistics for dichotomous outcomes. OR, HR, and 95% CI were calculated for each end point using a random effects or fixed model effects according to *I*^2^ value (if *I*^2^ value of <50%, we used fixed model effects). Subjects underwent early AVR and conservative care were defined as AVR group and conservative group, respectively. An OR/HR <1 favors early AVR and an OR/HR > 1 supports conservative management. Statistical heterogeneity was assessed by *I*^2^ statistic. *I*^2^ value of <50% indicated no obvious heterogeneity and *I*^2^ value of more than 50% indicated obvious heterogeneity. *P* values <0.05 were considered statistically significant and all values were two-sided. The adjusted HR was extracted if available from observational studies. If the HR was not described in a study, it was calculated from a Kaplan–Meier curve. Survival data was extracted using the Engauge Digitizer 10.8. Statistical analysis was performed using the Review Manager 5.3 (Nordic Cochrane Center, The Cochrane Collaboration, Copenhagen, Denmark) and survival analysis was performed with the IBM SPSS Statistics 26.

## Results

### Identification of Studies and Quality Assessment and Baseline Characteristics of Included Studies

**Figure 3:**
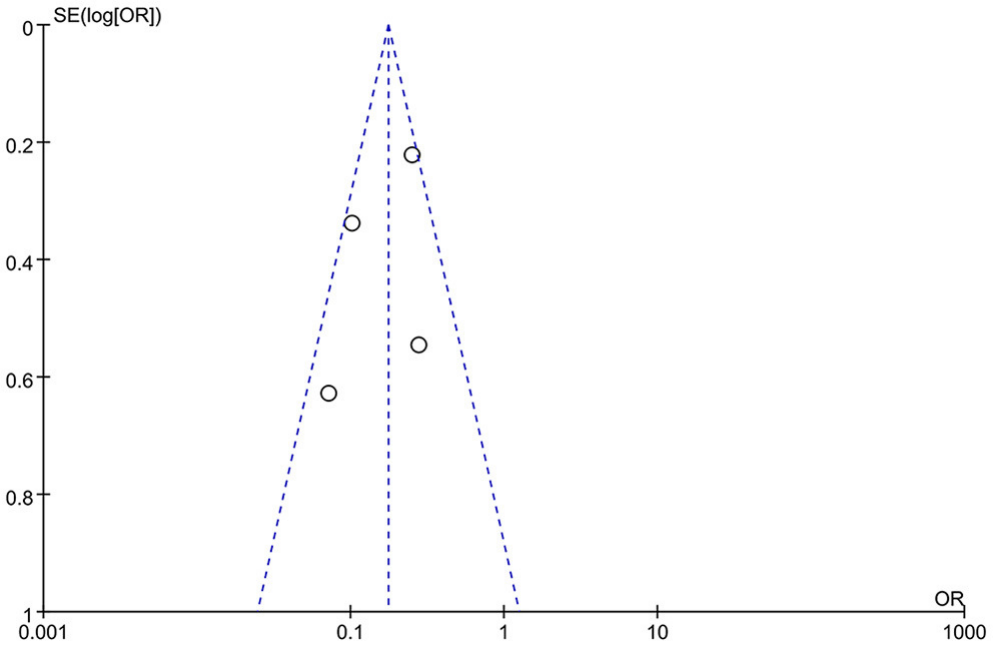
Funnel plot.

Our literature search yielded 2,478 studies. We acquired a total of 2,090 publications after the removal of duplicates. Fifteen articles were included for eligibility evaluation after careful screening of titles and abstracts. Eventually, four studies were included in this meta-analysis. The flow chart of literature search was shown in Figure 3. One eligible study was RCT, while the remaining three studies were prospective or retrospective cohort studies (Table 2). A total of 1,249 subjects with severe AS or very severe AS were included. Patients with asymptomatic severe and severe AS was assigned to the early AVR group (*n* = 588) or conservative strategy group (*n* = 661). The baseline characteristics of the four include articles were enclosed in Table 3. To assess the impact of patient selection on the pooled effect estimate, we performed a subgroup analysis, which exclusively included patients with very severe AS.

**Table 2:** Characteristics of the included trials.

**First author**	**Year**	**Study design**	**Region(s)**	**Study period**	**Number of patients**	**Follow-up (months)**
Kang	2020	RCT	South Korea	2010–2015	145	74.4 (60–88.8)^†^
						73.2 (54–87.6)^‡^
Kang	2010	Prospective	South Korea	1996–2006	197	42.2 (31.6–77.5)^†^
		cohort				59 (34–80.8)^‡^
Kim	2019	Retrospective cohort	South Korea	2000–2015	468	60.9 (29.9–107.0)
Bohbot	2018	Retrospective cohort	Europe, Multinational	2000–2015	439	60

*Age is expressed as mean ± SD*.

*Follow-up is reported as Median and IQR or mean ± SD*.

† *Early aortic valve replacement group*.

‡ *conservative management group*.

**Table 3:** Baseline characteristics of the patients of the included trials.

**Characteristic**	**Early AVR group (*****n*** **=** **588)**				**Conservative strategy group (*****n*** **=** **661)**			
	**Kang 2020**	**Kang 2010**	**Kim 2019**	**Bohbot 2018**	**Kang 2020**	**Kang 2010**	**Kim 2019**	**Bohbot 2018**
Age-y	65.0 ± 7.8	63 ± 11	61.0 ± 12.3	ND	63.4 ± 10.7	63 ± 11	67.1 ± 13.1	ND
Male gender-*n* (%)	37 (51)	55 (54)	110 (49.8)	ND	34 (47)	44 (46)	126 (51)	ND
Body surface area-m^2^	1.69 ± 0.17	1.65 ± 0.14	ND	ND	1.64 ± 0.17	1.62 ± 0.16	ND	ND
Body mass index	24.7 ± 3.4	23.9 ± 2.8	24.6 ± 3.2	ND	24.0 ± 2.6	24.1 ± 3.5	23.6 ± 3.2	ND
Diabetes-*n* (%)	13 (18)	10 (10)	37 (16.7)	ND	7 (10)	10 (11)	66 (26.7)	ND
Hypertension-*n* (%)	40 (55)	37 (36)	92 (41.6)	ND	39 (54)	39 (41)	122 (49.4)	ND
Smoking-*n* (%)	19 (26)	26 (25)	ND	ND	21 (29)	23 (24)	ND	ND
Hypercholesterolemia-*n* (%)	41 (56)	31 (30)	ND	ND	42 (58)	37 (39)	ND	ND
Coronary artery disease-*n*	5	ND	32	ND	1	ND	17	ND
Previous PCI-*n* (%)	3 (4)	ND	7 (3.2)	ND	1 (1)	ND	13 (5.3)	ND
Previous stroke-*n* (%)	3 (4)	ND	9 (4.1)	ND	3 (4)	ND	34 (13.8)	ND
Peripheral vascular disease-*n* (%)	1 (1)	ND	2 (0.9)	ND	2 (3)	ND	4 (1.6)	ND
Atrial fibrillation-*n* (%)	3 (4)	7 (7)	19 (8.6)	ND	6 (8)	8 (8)	34 (13.8)	ND
Serum creatinine level-mg/dl	0.84 ± 0.23	ND	0.9 ± 0.3	ND	0.83 ± 0.16	ND	1.1 ± 1.1	ND
EuroSCORE II score	0.9 ± 0.3^‡^	3.85 ± 1.66	ND	1.78 ± 0.92	0.9 ± 0.4^‡^	3.63 ± 1.92	ND	1.81 ± 0.93
Medication-*n* (%)								
ACEI	4 (5)	8 (8)	ND	ND	0	4 (4)	ND	ND
ARB	24 (33)	14 (14)	ND	ND	28 (39)	9 (9)	ND	ND
Calcium antagonist	19 (26)	9 (9)	ND	ND	20 (28)	13 (14)	ND	ND
Beta-blocker	13 (18)	19 (19)	ND	ND	8 (11)	12 (13)	ND	ND
Diuretic	13 (18)	15 (15)	ND	ND	17 (24)	13 (14)	ND	ND
Statin	34 (47)	ND	ND	ND	32 (44)	ND	ND	ND
Peak aortic jet velocity-m/sec	5.14 ± 0.52	ND	4.7 ± 0.7	ND	5.04 ± 0.44	ND	4.5 ± 0.6	ND
Transaortic pressure gradient-mm Hg								
Peak	106.9 ± 21.9	ND	ND	ND	102.5 ± 18.4	ND	ND	ND
Mean	64.3 ± 14.4	65 ± 13	55.0 ± 17.2	ND	62.7 ± 12.4	59 ± 12	48.6 ± 15.9	ND
Aortic valve area-cm^2^	0.63 ± 0.09	0.61 ± 0.10	0.74 ± 0.21	ND	0.64 ± 0.09	0.62 ± 0.09	0.80 ± 0.19	ND
Left ventricular mass index-g/m^2^	135.6 ± 38.2	158 ± 43	140.8 ± 54.4	ND	133.7 ± 31.1	159 ± 52	136.8 ± 40.3	ND
Left ventricular ejection fraction-%	64.8 ± 5.2	0.62 ± 0.07	63.7 ± 5.0	ND	64.8 ± 4.1	0.63 ± 0.07	63.1 ± 5.1	ND

*Age is expressed as mean ± SD*.

*ND, no data*.

*The body-mass index is the weight in kilograms divided by the square of the height in meters*.

‡ Scores on the European System for Cardiac Operative Risk Evaluation II (EuroSCORE II) are calculated by means of a logistic-regression equation and range from 0 to 100%.

*ACEI, Angiotensin-converting–enzyme inhibitor; ARB, Angiotensin-receptor blocker*.

### All-Cause Mortality

**Figure 4:**
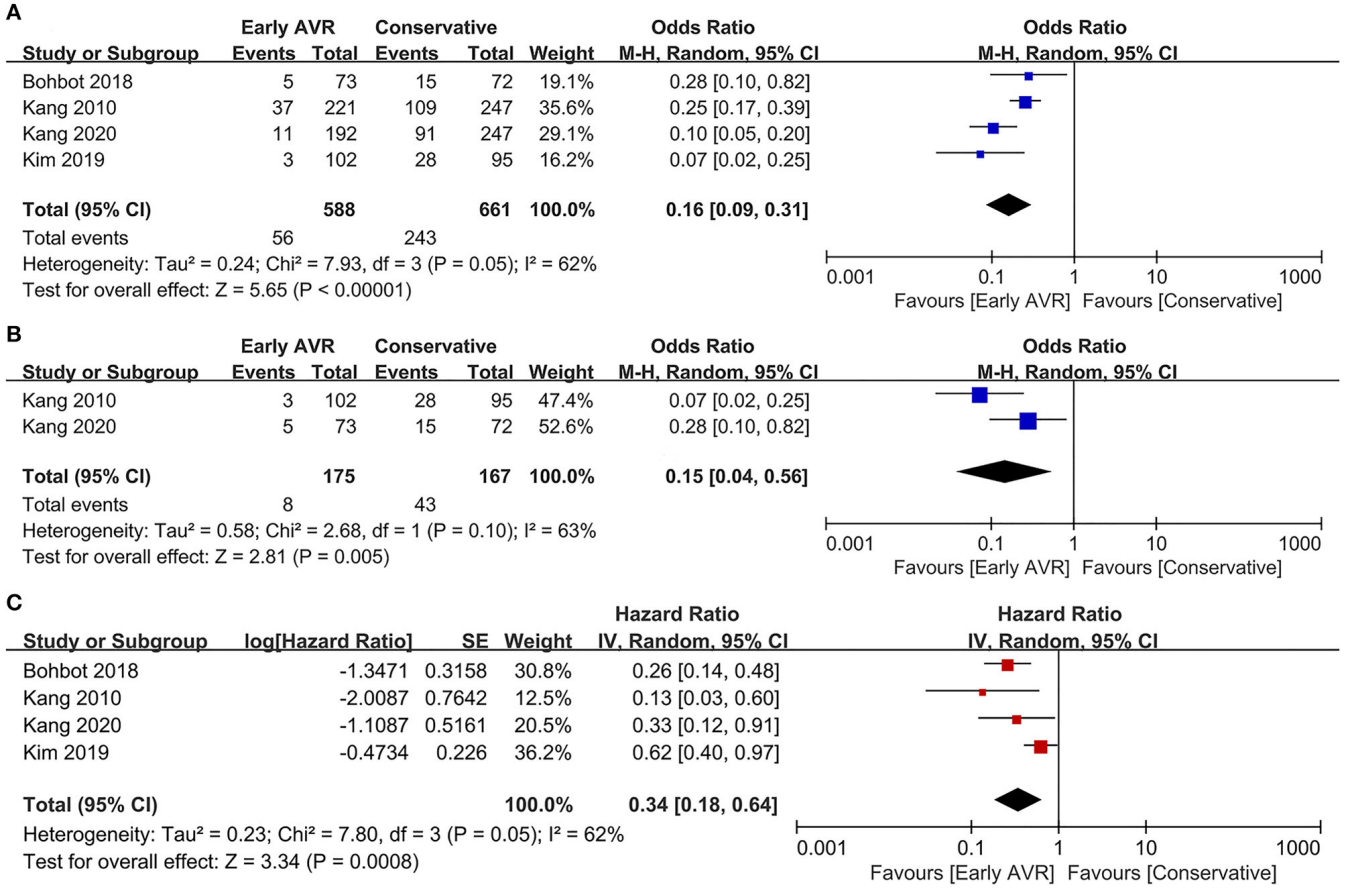
Assessment of the effect of early AVR on all-cause mortality. **(A,C)** Forrest plot of studies assessing the effect of early AVR and conservative strategy on all-cause mortality. **(B)** Forrest plot of studies assessing the effect of early AVR and conservative strategy in subjects with very severe AS on all-cause mortality.

Four studies (1, 12, 17, 18) (1,249 patients: early AVR group = 588; conservative strategy group = 661) included data of all-cause mortality. Early AVR was associated with significantly lower mortality compared to conservative care (OR 0.16, 95% CI 0.09–0.31, *P* < 0.00001, *I*^2^ = 62%; HR 0.34, 95% CI 0.18–0.64, *P* = 0.0008, *I*^2^ = 62%) (Figures 4A,C). Two studies only included very severe asymptomatic AS patients. There was also a remarkable reduction in all-cause mortality among early AVR subjects (OR 0.15, 95% CI 0.04–0.56, *P* = 0.005, *I*^2^ = 63%) (Figure 4B).

### Cardiac Mortality

**Figure 5:**
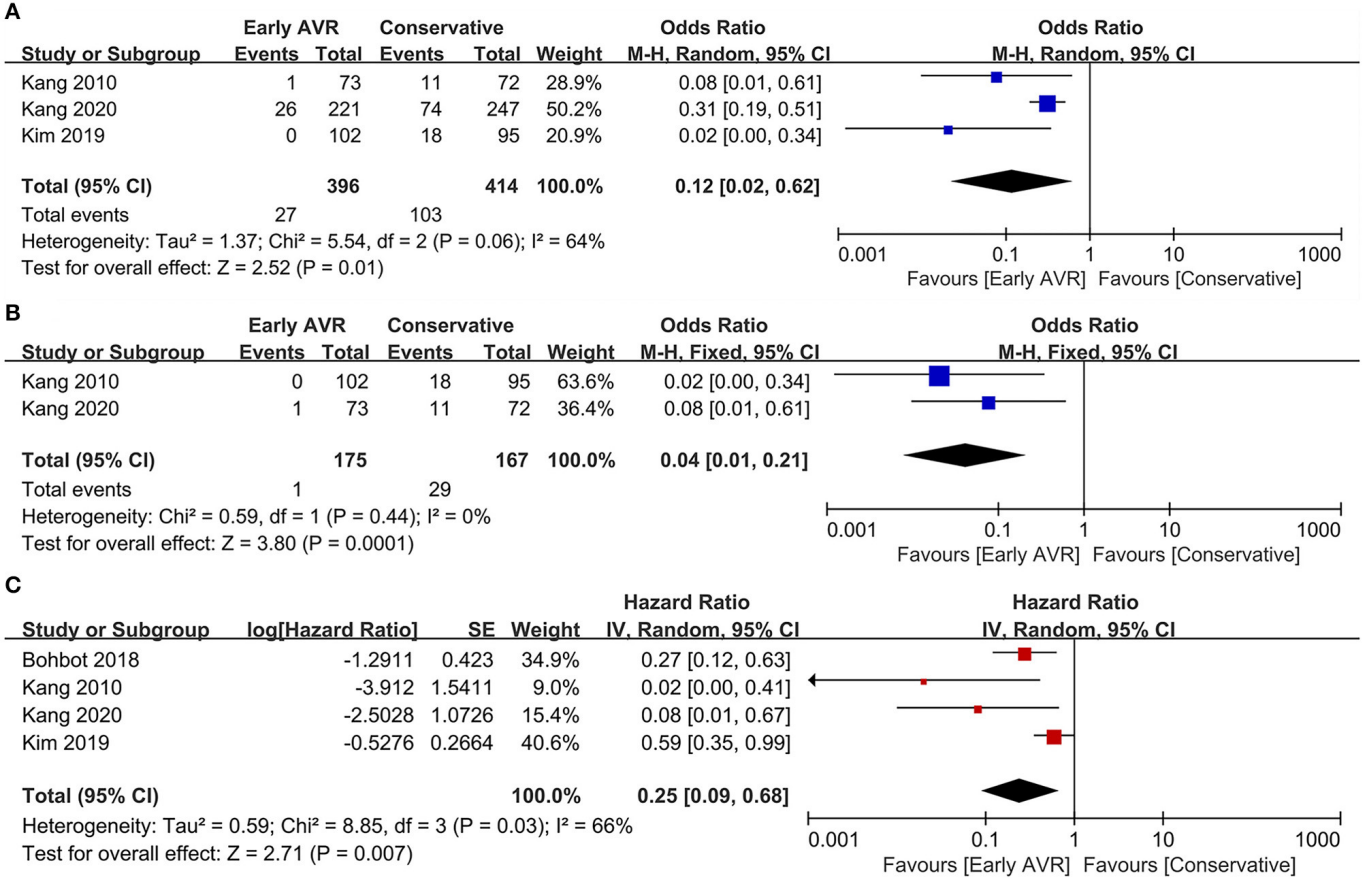
Assessment of the effect of early AVR on cardiac mortality. **(A,C)** Forrest plot of studies assessing the effect of early AVR and conservative strategy on cardiac mortality. **(B)** Forrest plot of studies assessing the effect of early AVR and conservative strategy in subjects with very severe AS on cardiac mortality.

Three studies (1, 17, 18) (810 patients: early AVR group = 396; conservative strategy group = 414) included data of cardiac mortality. Compared to conservative care group, early AVR was associated with significantly reduced cardiac mortality (OR 0.12, 95% CI 0.02–0.62, *P* = 0.01, *I*^2^ = 64%; HR 0.25, 95% CI 0.09–0.68, *P* = 0.007, *I*^2^ = 66%) (Figures 5A,C). For patients with very severe asymptomatic AS, there was also a significant reduction of cardiac mortality in early AVR group (OR 0.04, 95% CI 0.01–0.21, *P* = 0.0001, *I*^2^ = 0%) (Figure 5B).

### Non-cardiac Mortality

**Figure 6:**
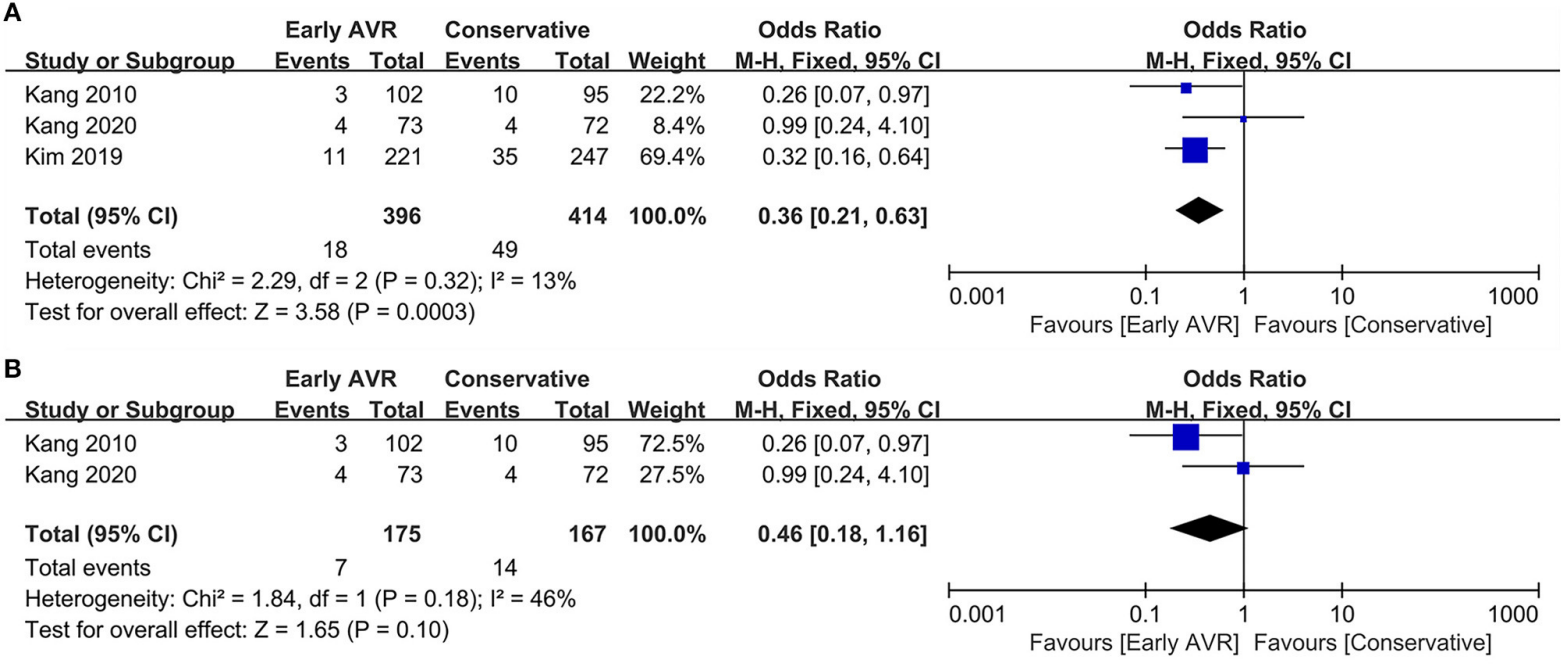
Assessment of the effect of early AVR on non-cardiac mortality. **(A)** Forrest plot of studies assessing the effect of early AVR and conservative strategy on non-cardiac mortality. **(B)** Forrest plot of studies assessing the effect of early AVR and conservative strategy in subjects with very severe AS on non-cardiac mortality.

Three studies (1, 17, 18) (810 patients: early AVR group = 396; conservative strategy group = 414) reported data of non-cardiac mortality. Compared to conservative care, early AVR significantly declined the non-cardiac mortality (OR 0.36, 95% CI 0.21–0.63, *P* = 0.0003, *I*^2^ = 13%) (Figure 6A). For patients with very severe asymptomatic AS, early AVR yielded no significant benefits over conservative care in non-cardiac mortality (OR 0.46, 95% CI 0.18–1.16, *P* = 0.10, *I*^2^ = 46%) (Figure 6B).

### Survival Analysis

**Figure 7:**
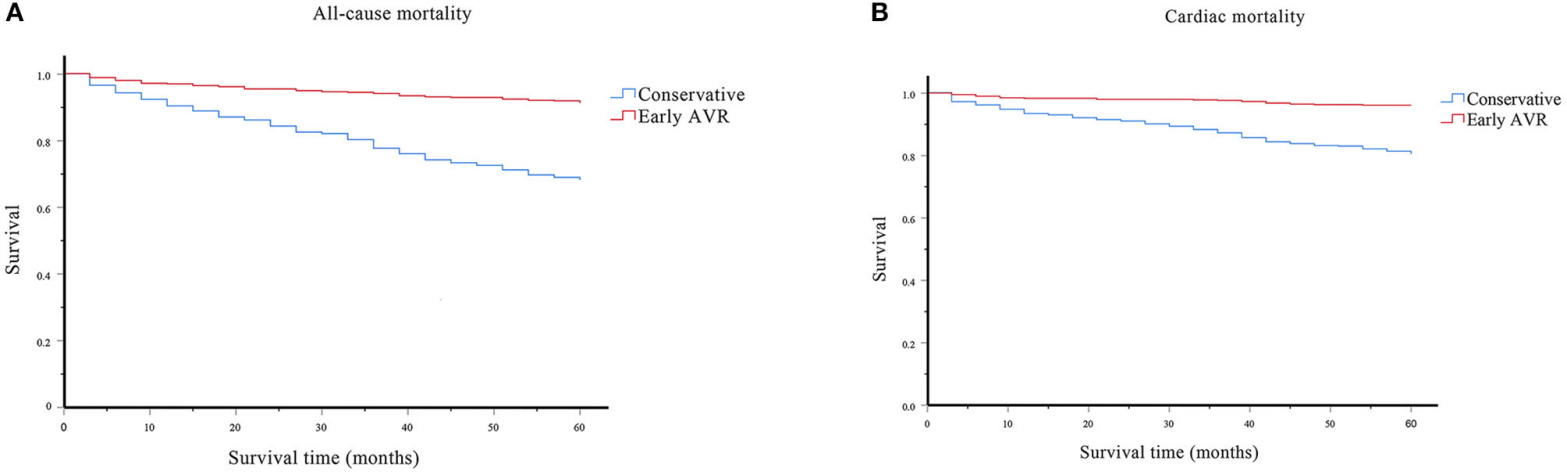
Survival assessment of the effect of early AVR on all-cause and cardiac mortality. **(A)** Survival analysis of all-cause mortality on subjects underwent early AVR or conservative strategy. **(B)** Survival analysis of cardiac mortality on subjects underwent early AVR or conservative strategy.

The cumulative overall survival rate, calculated with life table analysis, was 91% at 5 years in the early AVR group compared with 68% in the conservative-care group (*P* < 0.001) (Figure 7A). The 5 year survival rates (survival free of cardiac death) were 96 and 80% in the early AVR group and conservative-care group, respectively (*P* < 0.001) (Figure 7B).

### Comment

To the best of our knowledge, this is the first meta-analysis that compares the outcomes of early AVR procedure and conservative care approach in asymptomatic severe AS patients with preserved LVEF. Our analysis consists of 1,249 asymptomatic severe AS patients (LVEF ≥ 50%). In this meta-analysis, we conclude that early surgery is associated with a lower risk of all-cause mortality, cardiac mortality, and non-cardiac mortality.

A key predicament is whether the risk of AVR outweighs the risk of conservative treatment in patients of asymptomatic severe AS. Previous meta-analysis confirms an overall mortality reduction in AS patients after early AVR (19–21). These studies included patients with reduced LVEF, and did not exclude subjects who developed symptoms but deferred AVR procedure (19). Thus, we conducted this meta-analysis and demonstrate that asymptomatic severe AS patients with preserved LVEF could also benefit from early surgery. For patients with very severe asymptomatic AS, we draw the same conclusions considering its potential in reducing all-cause mortality and cardiac mortality. However, there is no significant difference in non-cardiac mortality. Moreover, we pooled the survival data from the studies. The pooled data indicate that early AVR reduces all-cause mortality in asymptomatic severe AS patients with preserved LVEF.

The risk stratification of patients with asymptomatic severe AS is controversial. Valvuloarterial impedance and left ventricular global longitudinal strain were deemed as sensitive markers to identify early AVR candidates (LVEF ≥ 50%) (22). Serum B-type natriuretic peptide (BNP) level, with a cut-off value of 100 pg/mL, was related to AS-related adverse events in asymptomatic patients (LVEF ≥ 50%) (23). Patients with BNP <100 pg/mL might benefit from watchful waiting strategy (23). However, the natriuretic peptides stratification had not been adapted in current clinical guidelines due to the lack of clinical evidence (24). In patients with asymptomatic AS (LVEF ≥ 50%), hs-TnT >10 ng/L was related with higher risk of events within 12 months (25). However, these findings were derived from observational studies and need to be confirmed in RCTs.

Our study had several limitations. First, the meta-analysis consists of only four studies, including three non-randomized trials that were subjected to possible selection bias. Moreover, two articles were from the same group. Second, not every end-point was reported in the four included studies. Third, there was not enough data about operative mortality. Consequently, we could not compare operative mortality. Fourth, we failed to include asymptomatic patients who underwent transcatheter aortic valve replacement (TAVR) due to limited data, which may be safer for high risk surgery candidates. Finally, we failed to obtain original survival data.

In summary, our meta-analysis suggests that early AVR is associated with reduced all-cause mortality, cardiac mortality, and non-cardiac mortality compared to conservative management in asymptomatic severe AS patients with preserved LVEF. More randomized controlled trials are currently underway (26–30). Based on the current findings in our meta-analysis, we tend to suggest clinicians to take an early interventional strategy for asymptomatic AS patients with preserved LVEF.

## Data Availability Statement

The original contributions presented in the study are included in the article/Supplementary Material, further inquiries can be directed to the corresponding author/s.

## Author Contributions

TY and YL collected data and performed analysis. TY and ZC wrote the manuscript. YL, CB, and ZC made revision of the manuscript. All authors contributed to the article and approved the submitted version.

## Conflict of Interest

The authors declare that the research was conducted in the absence of any commercial or financial relationships that could be construed as a potential conflict of interest.
