# Supplementary material for: Early Aortic Valve Replacement vs. Conservative Management in Asymptomatic Severe Aortic Stenosis Patients With Preserved Ejection Fraction: A Meta-Analysis
Source: Front Cardiovasc Med. 2021 Feb 3;7:621149. doi: 10.3389/fcvm.2020.621149 (PMC7887283; doi:10.3389/fcvm.2020.621149)
Supplement: Supplementary file 1 [file Data_Sheet_1.docx]

**Detailed searching strategy**

Pubmed:

((((((((((((((((("Asymptomatic Diseases"[Mesh]) OR (Disease, Asymptomatic[Title/Abstract])) OR (Diseases, Asymptomatic[Title/Abstract])) OR (Asymptomatic States[Title/Abstract])) OR (Asymptomatic State[Title/Abstract])) OR (Presymptomatic Diseases[Title/Abstract])) OR (Disease, Presymptomatic[Title/Abstract])) OR (Diseases, Presymptomatic[Title/Abstract])) OR (Presymptomatic Disease[Title/Abstract])) OR (Asymptomatic Conditions[Title/Abstract])) OR (Asymptomatic Condition[Title/Abstract])) OR (Condition, Asymptomatic[Title/Abstract])) OR (Conditions, Asymptomatic[Title/Abstract])) OR (Pre-Symptomatic Diseases[Title/Abstract])) OR (Disease, Pre-Symptomatic[Title/Abstract])) OR (Diseases, Pre-Symptomatic[Title/Abstract])) OR (Pre-Symptomatic Disease[Title/Abstract])) AND ((((((((("Aortic Valve Stenosis"[Mesh]) OR (Aortic Valve Stenoses)) OR (Stenoses, Aortic Valve)) OR (Stenosis, Aortic Valve)) OR (Valve Stenoses, Aortic)) OR (Valve Stenosis, Aortic)) OR (Aortic Stenosis)) OR (Stenoses, Aortic)) OR (Stenosis, Aortic))

Web of science:

1. TS=(Asymptomatic Disease  OR  Disease,  Asymptomatic  OR  Diseases,  Asymptomatic  OR  Asymptomatic  States  OR  Asymptomatic  State  OR  Presymptomatic  Diseases  OR  Disease,  Presymptomatic  OR  Diseases,  Presymptomatic  OR  Presymptomatic  Disease  OR  Asymptomatic  Conditions  OR  Asymptomatic  Condition  OR  Condition,  Asymptomatic  OR  Conditions,  Asymptomatic  OR  Pre-Symptomatic  Diseases  OR  Disease,  Pre-Symptomatic  OR  Diseases,  Pre Symptomatic  OR  Pre-Symptomatic  Disease)
2. TS=(Aortic stenosis  OR  Aortic  Valve  Stenoses  OR  Stenoses,  Aortic  Valve  OR  Stenosis,  Aortic  Valve  OR  Valve  Stenoses,  Aortic  OR  Valve  Stenosis,  Aortic  OR  Aortic  Stenosis  OR  Stenoses,  Aortic  OR  Stenosis,  Aortic)
3. 1 and 2

Embase：

Session Results

.......................................................

No. Query Results Date

#32. #21 AND #31 311 29 Jul 2020

#31. #22 OR #23 OR #24 OR #25 OR #26 OR #27 OR #28 OR 49,091 29 Jul 2020

#29 OR #30

#30. 'stenosis, aortic':ti,ab 518 29 Jul 2020

#29. 'stenoses, aortic':ti,ab 10 29 Jul 2020

#28. 'aortic stenosis':ti,ab 27,803 29 Jul 2020

#27. 'valve stenosis, aortic':ti,ab 51 29 Jul 2020

#26. 'valve stenoses, aortic':ti,ab 1 29 Jul 2020

#25. 'stenosis, aortic valve':ti,ab 202 29 Jul 2020

#24. 'stenoses, aortic valve':ti,ab 3 29 Jul 2020

#23. 'aortic valve stenoses':ti,ab 24 29 Jul 2020

#22. 'aortic stenosis'/exp 45,604 29 Jul 2020

#21. #3 OR #5 OR #6 OR #7 OR #8 OR #9 OR #10 OR #11 OR 15,621 29 Jul 2020

#12 OR #13 OR #14 OR #15 OR #16 OR #17 OR #18 OR

#19 OR #20

#20. 'pre-symptomatic disease':ti,ab 65 29 Jul 2020

#19. 'diseases, pre-symptomatic':ti,ab 1 29 Jul 2020

#18. 'disease, pre-symptomatic':ti,ab 7 29 Jul 2020

#17. 'pre-symptomatic diseases':ti,ab 29 Jul 2020

#16. 'conditions, asymptomatic':ti,ab 15 29 Jul 2020

#15. 'condition, asymptomatic':ti,ab 15 29 Jul 2020

#14. 'asymptomatic condition':ti,ab 358 29 Jul 2020

#13. 'asymptomatic conditions':ti,ab 95 29 Jul 2020

#12. 'presymptomatic disease':ti,ab 101 29 Jul 2020

#11. 'diseases, presymptomatic':ti,ab 2 29 Jul 2020

#10. 'disease, presymptomatic':ti,ab 32 29 Jul 2020

#9. 'presymptomatic diseases':ti,ab 4 29 Jul 2020

#8. 'asymptomatic state':ti,ab 312 29 Jul 2020

#7. 'asymptomatic states':ti,ab 24 29 Jul 2020

#6. 'diseases, asymptomatic':ti,ab 11 29 Jul 2020

#5. 'disease, asymptomatic':ti,ab 196 29 Jul 2020

#3. 'asymptomatic disease'/exp 14,626 29 Jul 2020

Cochrane library:

#1 Mesh descriptor: [Asymptomatic Disease] explode all trees

#2 (Disease, Asymptomatic): ti,ab,kw OR (Diseases, Asymptomatic): ti,ab,kw OR (Asymptomatic States): ti,ab,kw OR (Asymptomatic State): ti,ab,kw OR (Presymptomatic Diseases): ti,ab,kw

#3 (Disease, Presymptomatic): ti,ab,kw OR (Diseases, Presymptomatic): ti,ab,kw OR (Presymptomatic Disease): ti,ab,kw OR (Asymptomatic Conditions): ti,ab,kw OR (Asymptomatic Condition): ti,ab,kw

#4 (Condition, Asymptomatic): ti,ab,kw OR (Conditions, Asymptomatic): ti,ab,kw OR (Pre-symptomatic Diseases): ti,ab,kw OR (Disease, Pre-symptomatic): ti,ab,kw OR (Diseases, Pre-symptomatic) : ti,ab,kw

#5 (Pre-symptomatic Disease): ti,ab,kw

#6 #1 or #2 or #3 or #4 or #5

#7 Mesh descriptor: [Aortic Valve Stenosis] explode all trees

#8 (Aortic Valve Stenoses): ti,ab,kw OR (Stenoses, Aortic Valve): ti,ab,kw OR (Stenosis, Aortic Valve): ti,ab,kw OR (Valve Stenoses, Aortic): ti,ab,kw OR (Valve Stenosis, Aortic): ti,ab,kw

#9 (Aortic Stenosis): ti,ab,kw OR (Stenoses, Aortic): ti,ab,kw OR (Stenosis, Aortic): ti,ab,kw

#10 #7 or #8 or#9

#11 #6 and #10
